# Polar Lipid Fraction E from *Sulfolobus acidocaldarius* and Dipalmitoylphosphatidylcholine Can Form Stable yet Thermo-Sensitive Tetraether/Diester Hybrid Archaeosomes with Controlled Release Capability

**DOI:** 10.3390/ijms21218388

**Published:** 2020-11-09

**Authors:** Umme Ayesa, Parkson Lee-Gau Chong

**Affiliations:** Department of Medical Genetics and Molecular Biochemistry, Lewis Katz School of Medicine at Temple University, Philadelphia, PA 19140, USA; tua46021@temple.edu

**Keywords:** archaeosomes, tetraether lipids, thermosensitive liposomes, controlled release, mild hyperthermia, doxorubicin, zeta potential, fluorescent probes

## Abstract

Archaeosomes have drawn increasing attention in recent years as novel nano-carriers for therapeutics. The main obstacle of using archaeosomes for therapeutics delivery has been the lack of an efficient method to trigger the release of entrapped content from the otherwise extremely stable structure. Our present study tackles this long-standing problem. We made hybrid archaeosomes composed of tetraether lipids, called the polar lipid fraction E (PLFE) isolated from the thermoacidophilic archaeon *Sulfolobus acidocaldarius*, and the synthetic diester lipid dipalmitoylphosphatidylcholine (DPPC). Differential polarized phase-modulation and steady-state fluorometry, confocal fluorescence microscopy, zeta potential (ZP) measurements, and biochemical assays were employed to characterize the physical properties and drug behaviors in PLFE/DPPC hybrid archaeosomes in the presence and absence of live cells. We found that PLFE lipids have an ordering effect on fluid DPPC liposomal membranes, which can slow down the release of entrapped drugs, while PLFE provides high negative charges on the outer surface of liposomes, which can increase vesicle stability against coalescence among liposomes or with cells. Furthermore, we found that the zeta potential in hybrid archaeosomes with 30 mol% PLFE and 70 mol% DPPC (designated as PLFE/DPPC(3:7) archaeosomes) undergoes an abrupt increase from −48 mV at 37 °C to −16 mV at 44 °C (termed the ZP transition), which we hypothesize results from DPPC domain melting and PLFE lipid ‘flip-flop’. The anticancer drug doxorubicin (DXO) can be readily incorporated into PLFE/DPPC(3:7) archaeosomes. The rate constant of DXO release from PLFE/DPPC(3:7) archaeosomes into Tris buffer exhibited a sharp increase (~2.5 times), when the temperature was raised from 37 to 42 °C, which is believed to result from the liposomal structural changes associated with the ZP transition. This thermo-induced sharp increase in drug release was not affected by serum proteins as a similar temperature dependence of drug release kinetics was observed in human blood serum. A 15-min pre-incubation of PLFE/DPPC(3:7) archaeosomal DXO with MCF-7 breast cancer cells at 42 °C caused a significant increase in the amount of DXO entering into the nuclei and a considerable increase in the cell’s cytotoxicity under the 37 °C growth temperature. Taken together, our data suggests that PLFE/DPPC(3:7) archaeosomes are stable yet potentially useful thermo-sensitive liposomes wherein the temperature range (from 37 to 42–44 °C) clinically used for mild hyperthermia treatment of tumors can be used to trigger drug release for medical interventions.

## 1. Introduction

Conventional chemotherapeutic drugs such as doxorubicin (DXO) are highly effective. However, they have short half-lives and cause severe side effects like cardio and gastrointestinal toxicity [1,2]. To minimize non-specific cytotoxicity, liposomes have been used clinically for drug delivery [3,4]. Additionally, tumor tissues and nearby blood vasculatures have enhanced permeability and retention, which allow liposomes to accumulate [5,6,7]. Various types of liposomal doxorubicin, such as Doxil, Caelyx, and Myocet, have been demonstrated to reduce systemic toxicity, prolong circulation time, and increase accumulation at tumor sites [8,9,10]. However, they have major drawbacks including low stability and slow drug release at physiological conditions [11,12]. Moreover, liposomes that accumulate at tumor site often fail to penetrate deep into tumor tissue, rendering the drug therapeutic efficacy low [13,14].

One way to improve drug efficacy is by inducing drug release from thermo-sensitive liposomes (TSL) [15] into the tumor by treating the tumor site with local mild hyperthermia (i.e., raising the local body temperature from 37 °C to 42-44 °C) [13,16], which is normally controlled to below 45 °C to avoid vascular hemorrhage. In addition, hyperthermia improves blood flow and permeability near tumor vessels, increasing nanoparticle extravasation [14,17]. Several thermo-sensitive liposomes have been formulated to attain maximum drug release in response to hyperthermia treatment [18,19,20,21,22,23,24,25,26,27,28,29]. One thermo-sensitive liposome currently in phase III clinical trials is ThermoDox. ThermoDox formulation contains exclusively diester lipids including 1,2-dipalmitoyl-*sn*-glycero-3-phosphocholine (DPPC), with a main phase transition temperature (T_m_) of 41.5 °C, a single chain lysolipid monostearoylphosphatidylcholine (MSPC), and 1,2-distearoyl-*sn*-glycero-3-phosphoethanolamine-N-[methoxy(poly(ethylene glycol))-2000] (DSPE-PEG-2000) [18,30]. MSPC is used to slightly lower the T_m_ of DPPC allowing drug release to occur with mild hyperthermia [11,31]. A caveat of adding MSPC to liposomal formulation is that MSPC makes the liposomal system unstable at physiological temperature [32]. To increase liposomal stability, cholesterol and PEG-conjugated lipids have been included in the formulation. However, cholesterol can resist thermal shock by stabilizing the gel state of the lipid membrane [15,33], while the polyethylene glycol moiety reduces drug loading at higher concentrations, leading to decreased drug exposure at the tumor site [29,32,34].

In the present study, we used archaea bipolar tetraether lipids, specifically, the polar lipid fraction E (PLFE, Figure S1) extracted from the thermoacidophilic archaeon *Sulfolobus acidocaldarius* as the stabilizing agent to make DPPC-based thermo-sensitive liposomes. PLFE is a mixture containing two types of hydrophobic cores, i.e., GDNT (glycerol dialkylcalditol tetraether) and GDGT (glycerol dialkyglycerol tetraether) (Figure S1) [35,36]. The GDNT component (~90% of total PLFE) has phospho-*myo*-inositol on the glycerol end and β-d-glucose on the calditol end. The GDGT component (~10%) contains phospho-*myo*-inositol linked to one glycerol and β-d-galactosyl-d-glucose to the other glycerol skeleton (Figure S1). The hydrophobic region of PLFE consists of a pair of 40-carbon biphytanyl chains, each of which has isoprene units and may include up to four cyclopentane rings. PLFE liposomes are extraordinarily stable, compared to liposomes made of diester lipids. PLFE liposomal membranes are low in permeability, compressibility, and volume fluctuations [37,38,39,40,41]. In addition, PLFE liposomes show high resistance to autoclaving, acidic environment, and fusogenic compounds [42,43,44]. PLFE liposomes exhibit phase transitions, which is not common for archaea lipids; however, the enthalpy and volume changes associated with the PLFE phase transitions are unusually low [45,46]. These characteristics of PLFE liposomes can be attributed to the tight and rigid membrane packing [47] due to the presence of tetraether linkages and cyclopentane rings in the dibiphytanyl chains as well as an extensive hydrogen bond network engendered by the sugar and phosphate moieties exposed at the liposome surface [48,49,50,51].

In this work, we have characterized the physical properties of PLFE/DPPC archaeosomes as a function of membrane composition and temperature. We found that PLFE stabilizes the liposomal membranes, similar to the results obtained from other tetraether lipids [52,53,54]. However, surprisingly, in contrast to tetraether archaeosomes previously studied by others, our hybrid archaeosomes at a particular PLFE/DPPC molar ratio were found to undergo a dramatic change in zeta potential when the temperature was raised from 37 °C to 44 °C. This temperature elevation coincides with the temperature range used clinically for mild hyperthermia treatment of tumors. We have, therefore, studied the effects of hyperthermia temperature jump on the interactions of PLFE/DPPC archaeosomal DXO with live cells by using biochemical assays and confocal fluorescence microscopy. We found that such a mild temperature perturbation can significantly increase DXO release, the entry of DXO into the nuclei of target cells, and the drug’s cytotoxicity. These results show that PLFE/DPPC, a tetraether–diester hybrid archaeosome, is not only extremely stable, but can also be tailored to be thermo-sensitive nano-carriers potentially useful for various medical intervention applications.

## 2. Results and Discussions

### 2.1. Effect of PLFE Molar Content on Zeta Potential of DPPC/PLFE Hybrid Archaeosomes

PLFE contains exclusively bipolar tetraether lipids [35]. When forming liposomes, PLFE lipids span the entire membrane generating a monomolecular structure [45] whereas DPPC, a diester lipid, forms a bilayer when dispersed in an aqueous solution. Thus, liposomes made of PLFE and DPPC should have monolayer/bilayer hybrid structures. While monolayer liposomes made of PLFE alone or bilayer liposomes made of DPPC alone have been studied extensively [41,47], PLFE/DPPC hybrid archaeosomes have not yet been characterized to a great extent.

**Figure 1:**
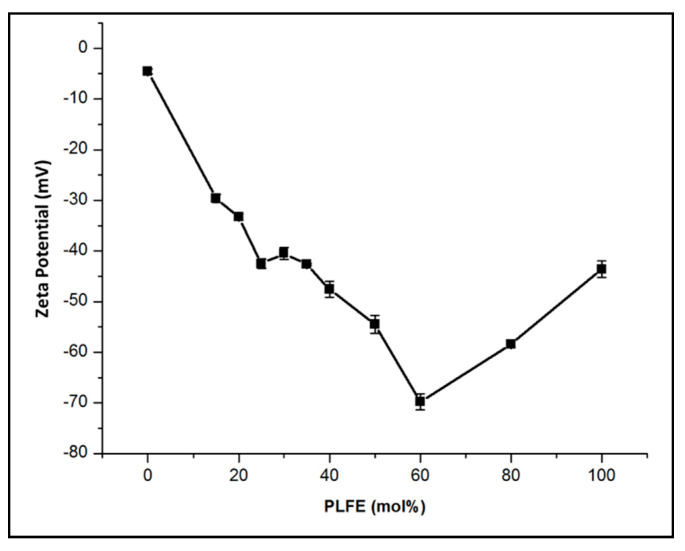
Effect of polar lipid fraction E (PLFE) mole fraction on the zeta potential (ZP) of PLFE/dipalmitoylphosphatidylcholine (DPPC) hybrid archaeosomes. Buffer: 50 mM Tris buffer (pH 7.2) containing 10 mM EDTA and 0.02% NaN_3_. Temperature: ~22 °C. Liposome size: 145-163 nm in diameter. Polydispersity: < 0.2. Error bars are the standard deviations from three measurements.

In order to determine the stability of PLFE/DPPC hybrid archaeosomes in solution, zeta potential (ZP) was measured as a function of PLFE mole fraction. ZP of liposome is the electric potential at the slipping plane of the interfacial double layer that exists on the surface of a particle in solution. Thus, it is not the same as the surface potential of liposomes. However, when the electrolytes and ionic strength in the liposome solution remain constant, the change in ZP reflects the change in membrane surface charge. It is generally accepted that a ZP value of ≤ −30 mV or ≥ +30 mV would indicate high stability against vesicle coalescence due to charge–charge repulsion [55,56]. The ZP value of DPPC liposomes in 50 mM Tris buffer (pH 7.2) containing 10 mM ethylenediaminetetraacetic acid (EDTA) and 0.02% NaN_3_ was measured to be −4.6 ± 0.5 mV (Figure 1). In the same buffer, with an increase in the mole fraction of PLFE, the ZP value of PLFE/DPPC archaeosomes became more negative, reaching the most negative value −69.8 ± 1.8 mV at 60 mol% PLFE. Beyond 60 mol% PLFE, this trend was reversed, forming a biphasic change in ZP with PLFE mole fraction (Figure 1). The ZP value of liposomes at 100% PLFE is −42 mV (Figure 1), in good agreement with the value previously reported [37]. It can be concluded from Figure 1 that PLFE/DPPC hybrid archaeosomes with PLFE content greater than 15 mol% are highly stable because their ZP values are more negative than −30 mV.

PLFE lipids are asymmetric, macrocyclic molecules, each of which has two different polar head groups (Figure S1). The inositol end of PLFE lipids contains a phosphate, which is negatively charged at neutral pH, whereas the other polar end of PLFE lipids carries a net charge of zero. Thus, the surface charge of PLFE/DPPC archaeosomes depends on PLFE content and the orientation of PLFE lipids in the liposomal membrane. This explains why ZP can become more negative when PLFE mol% in the hybrid archaeosomes increases from 0 to 60 mol% (Figure 1). It is possible that, at 60 mol% PLFE, the charge–charge repulsion among the phospho-inositol moieties on PLFE at the liposome outer surface reaches a limit. As a result, the addition of more PLFE (> 60 mol%) causes PLFE lipid inversion bidirectionally (termed flip-flop) across the membrane resulting in a significant change in the ratio of the charge on the outer surface (Q1) to that on the inner surface (Q2). A recent theoretical study suggests that the ions in the slipping plane may be affected by the charge on both the outer and the inner surface of the liposome [57]. Hence, a major change in Q1 relative to Q2 could cause a biphasic change in ZP with PLFE content, as shown in Figure 1.

### 2.2. Thermo-Induced ZP Transition 

**Figure 2:**
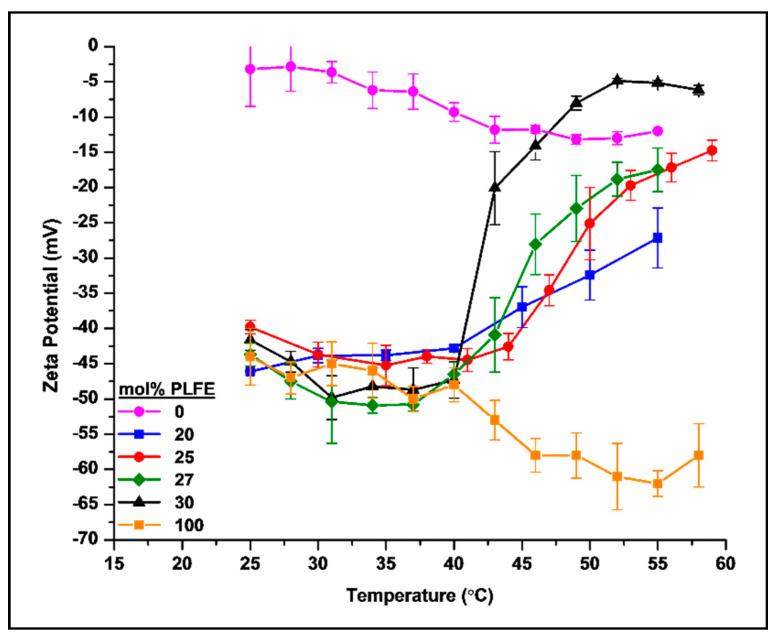
Temperature dependence of ZP measured on PLFE/DPPC liposomes at various PLFE mole fractions over the temperature range 25–58 °C. Error bars are the standard deviations from three independent measurements. Particle size (146 ± 2.5 nm) and polydispersity (0.09 ± 0.01) of PLFE/DPPC(3:7) archaeosomes remained virtually unchanged in the temperature range examined. Liposomes were dispersed in 50 mM Tris buffer (pH 7.2) containing 10 mM EDTA and 0.02% NaN_3_.

We have examined how temperature affects the ZP in PLFE/DPPC mixtures. As shown in Figure 2, there is an abrupt increase in zeta potential (called the ZP transition) with increasing temperature for DPPC liposomes containing 25–30 mol% PLFE. For 25 mol% PLFE/75 mol% DPPC, the abrupt increase occurred at ~47.5 °C (red circles, Figure 2). For 27 mol% PLFE/73 mol% DPPC, the middle point of the ZP transition occurred at ~45 °C (green diamonds, Figure 2). The most striking ZP transition comes from liposomes composed of 30 mol% PLFE and 70 mol% DPPC (designated as PLFE/DPPC(3:7) archaeosomes). At this mole fraction, ZP remains steadily negative at 25–37 °C. Thereafter, ZP undergoes a dramatic change from −48 mV at 37 °C to −16 mV at 44 °C, with the most abrupt change at ~42 °C, followed by a leveling off at 50–58 °C (black triangles, Figure 2).

The ZP transition obtained from a heating scan appears to be reversible upon cooling (scan rate ~0.2 °C/min); however, hysteresis is noticed (Figure S2), indicating that the ZP transition is indeed due to physical changes in the membrane structure and that it needs a much longer time than we used in the ZP scan to reach the thermodynamic equilibrium of the structural changes. It is also found that, for PLFE/DPPC archaeosomes containing more than 30 mol% PLFE, ZP just decreases steadily, lacking an abrupt change, with increasing temperature, as illustrated in Figure 2 (orange squares) for the case of 100 mol% PLFE.

The large and abrupt ZP change with a mild temperature perturbation obtained from PLFE/DPPC(3:7) archaeosomes is a novel finding. To the best of our knowledge, this phenomenon has not been reported in other liposome systems. Since the ZP value reflects membrane surface charge, our data suggests that, in PLFE/DPPC(3:7) archaeosomes, the surface charge undergoes a dramatic change from very negative to much less negative when the temperature is changed from the body temperature (37 °C) to 44 °C. We speculate that this dramatic change in ZP is caused by flip-flopping of PLFE lipids across the membrane, triggered by the melting of DPPC domains at ~41.5 °C, the main phase transition temperature of DPPC. Note that PLFE lipids are negatively charged at neutral pH only at one of the two polar head groups. It is possible that the negatively charged polar head group of PLFE flips from the outer surface to the inner surface of the liposome, giving rise to a significant change in the charge on the outer surface relative to the charge on the inner surface, consequently resulting in a ZP transition.

We attribute the ZP transition to the presence of DPPC domains because the temperature dependence of generalized polarization (GP) of 6-lauroyl-1,2-dimethylamino-naphthalene (Laurdan) fluorescence shows an abrupt change at or close to the phase transition temperature of DPPC for PLFE/DPPC mixtures with 0–30 mol% PLFE (Figure S3). An abrupt change in Laurdan’s GP is indicative of domain formation. The lack of an abrupt change in Laurdan’s GP for PLFE/DPPC with more than 30 mol% PLFE (Figure S3) is consistent with the observation that the ZP transition was not observed at > 30 mol% PLFE (Figure 2). However, the melting of the gel phase of DPPC domains alone does not explain the dramatic ZP transition appeared in PLFE/DPPC(3:7) archaeosomes because 100 mol% DPPC does not show any abrupt change in ZP with temperature. Instead, the ZP value of 100 mol% DPPC just decreases monotonically with temperature (purple circles, Figure 2), which agrees with the previous studies and can be attributed to changes in the orientation of the phosphatidylcholine polar head groups with respect to the membrane surface [58]. Furthermore, we propose that, in conjunction with the DPPC domain melting, the mild temperature perturbation from 37 to 44 °C causes PLFE lipid flip-flop, leading to the ZP transition. Lin et al. previously showed that lipid flip-flop happened most frequently at the interface between symmetric and asymmetric domains [59]. In our case, DPPC domains are symmetric longitudinally across the membrane, whereas PLFE domains are asymmetric. PLFE lipid flip-flop may occur in the domain interfacial regions, especially during the time of DPPC domain melting.

More detailed biophysical investigations are needed in the future studies in order to further elucidate the mechanism underlying the ZP transition. Our current focus is to assess whether the sharp ZP transition detected in PLFE/DPPC(3:7) archaeosomes can be used to develop a new type of thermo-sensitive nano-carriers of therapeutic agents. We believe that this is highly plausible because the sharp ZP transition in this liposomal formulation occurs at the temperature range that is clinically used for mild hyperthermia treatment (37 to 42--44 °C) of tumors. Using the existing technologies (such as ultrasound) for hyperthermia treatment, we can locally heat the tumor areas to induce the ZP transition and consequently increase drug release from the hybrid archaeosomes that are at or near the tumors. In addition, before the ZP transition, PLFE/DPPC(3:7) archaeosomes are highly negatively charged and, as a result, there will be little interaction among themselves and between PLFE/DPPC(3:7) archaeosomes and cancer cells as most cancer cells are negatively charged on the extracellular surface. After the ZP transition, the archaeosome surface becomes much less negatively charged. This change in liposome surface charge may uniquely facilitate liposome interaction with the negative surface of cancer cells.

It is important to mention that not all strong anionic (negatively charged) liposomes are prone to the reticulo-endothelial system (RES) uptake. The stability of anionic liposomes in the circulation is dependent on liposome compositions [60,61]. Anionic liposomes without sugar moieties on the surface are prone to RES attacks and preferentially taken up by macrophages [62]. However, negatively charged liposomes containing gangliosides are stable in the serum and have long circulation times [63,64]. PLFE lipids are like gangliosides in that they have multiple sugar moieties in the polar headgroups, which form extensive hydrogen bond networks on the liposome surface. Such a hydrophilic layer would hinder the binding of blood proteins such as immunoglobulins and opsonins, consequently reducing phagocytic cell uptake [65]. Thus, the high negative surface charge does not necessarily limit therapeutic utility of our archaeosomes.

### 2.3. Membrane Dynamics of PLFE/DPPC(3:7) Archaeosomes as Explored by DPH Nanosecond Fluorometry

Since PLFE/DPPC(3:7) archaeosomes exhibit a distinct ZP transition and are potential thermo-sensitive liposomes for drug delivery, it is of interest to further characterize their physical properties. We have used the differential polarized phase-modulation fluorometry [66,67] to study the order parameter (S) and the rotational rate (R) experienced by the membrane probe 1,6-diphenyl-1,3,5-hexatriene (DPH) in PLFE/DPPC(3:7) hybrid archaeosomes, as compared to DPPC and PLFE liposomes.

The measured differential phase delay and demodulation ratio between the parallel and perpendicular component of the DPH emission were fitted by various anisotropy (r) decay laws. It was judged from the reduced χ^2^ that the two-exponential decay equation r(t) = r_1_exp(-t/θ_1_) + r_2_exp(-t/θ_2_), with θ_2_ (the rotational correlation time of component 2) fixed to a large number such as 10,000 ns, best fits the data. In this case, r_2_ is essentially the limiting anisotropy r_∞_, which is the lowest value of anisotropy attainable at times that are long compared to the fluorescence lifetime [68]. Thus, the anisotropy decay of DPH in the three membrane systems examined is best described by the equation: r(t) = r_1_exp(-t/θ_1_) + r_∞_. The fitted parameters, such as the limiting anisotropy r_∞_, the rotational correlation time θ_1_, and the rotational rate (R) for DPH in DPPC, PLFE, and PLFE/DPPC(3:7) archaeosomes at various temperatures are listed in Table S1. The rotational rate was calculated using the equation R = 1/6θ_1_ [69]. r_∞_ reflects the rotational hindrance of the probe. In an isotropic solvent without any rotational hindrance, r_∞_ should be close to zero. Conversely, in a tightly packed lipid membrane, r_∞_ is significantly higher than zero. The order parameter (S) was calculated by the equation: S^2^ = r_∞_/r_0_ [70], where r_0_ **=** 0.39 is the fundamental anisotropy of DPH observed in the absence of rotational motion during lifetime of excited state [71].

**Figure 3:**
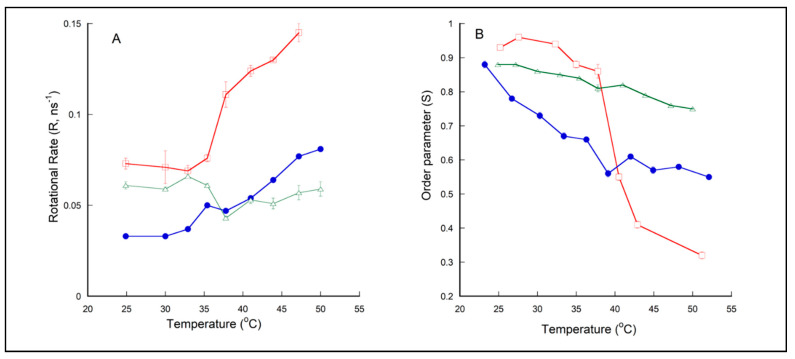
Temperature dependence of (**A**) the rotational rate constant, R, and (**B**) order parameter, S, experienced by 1,6-diphenyl-1,3,5-hexatriene (DPH) in liposomal membranes made of DPPC (red squares), PLFE (green triangles), or PLFE/DPPC(3:7) archaeosomes (blue circles). Particle size range: 154–160 nm. Error bars are the standard deviations from three separated measurements. Comparing the S value at 39.1 °C with that at 36.3 °C or 42.0 °C for PLFE/DPPC(3:7) archaeosomes, gives a *p* value < 0.01.

Figure 3B shows that the order parameter (S) for DPPC liposomes (red squares) decreases dramatically from ~0.90–0.95 at 25–35 °C (gel state of DPPC) to ~0.25 at 55 °C (liquid–crystalline state of DPPC), with the mid-point of the abrupt change occurred at ~41–42 °C, which matches with the T_m_ value of DPPC. The corresponding r_∞_ values, i.e., 0.30–0.34 at 25–35 °C and 0.048 at 55 °C (Table S1), are in excellent agreement with the r_∞_ values of DPH in DPPC liposomes previously reported (0.33 for gel-state DPPC and 0.05 for liquid–crystalline DPPC, [72]). Figure 3A shows that the R value of DPH in DPPC liposomes (red squares) undergoes an abrupt increase with increasing temperature near the T_m_ of DPPC, a trend also fully consistent with previously reported [72].

The DPH anisotropy decay data obtained from PLFE and PLFE/DPPC(3:7) archaeosomes are new findings. The S value of PLFE liposomes decreases moderately and monotonically and remains relatively high with increasing temperature, from 0.88 at 24.9 °C to 0.75 at 50.0 °C (Figure 3B, green triangles). This result echoes the previous finding that PLFE liposomes are rigid and tightly packed over a wide range of temperatures [73]. Figure 3B also shows that, at temperatures below the DPPC main phase transition temperature (41.5 °C), the order parameter of DPH in PLFE liposomes is slightly lower than that in gel-state DPPC; however, at temperatures above 41.5 °C, the order parameter in PLFE liposomes is much higher than that in liquid–crystalline state DPPC. At temperatures below 41.5 °C, the rotational rate (R) of DPH in PLFE liposomes is comparable to that in gel-state DPPC. At temperatures > 41.5 °C, R in PLFE liposomes is much lower than that in the liquid–crystalline state DPPC (Figure 3A). It is clear from these DPH fluorescence data that membrane dynamic structures in PLFE liposomes and gel-state DPPC are similar; however, there is a considerable difference in the dynamic structure between fluid state DPPC and PLFE liposomes at the respective temperatures.

More interesting is the temperature dependence of R and S in PLFE/DPPC(3:7) archaeosomes. PLFE/DPPC(3:7) archaeosomes have S values lower than those in gel-state DPPC but higher than those in fluid state DPPC at the respective temperatures. This suggests that PLFE lipids loosen membrane packing of gel-state DPPC but tighten membrane packing of fluid state DPPC. A similar effect occurs in lipid membranes containing cholesterol, a frequently used stabilizing agent in liposomal drug formulation [12,74]. Thus, from the membrane ordering point of view, PLFE lipids could also be used as a stabilizing agent in liposomal drugs.

Above 40–41 °C, as expected from a random mixture of PLFE and DPPC, the R and S values of PLFE/DPPC(3:7) archaeosomes lie between those of DPPC and PLFE liposomes (Figure 3). However, to our surprise, below 40–41 °C, R and S of DPH in PLFE/DPPC(3:7) archaeosomes are significantly lower than those in DPPC and PLFE liposomes (Figure 3). One possible explanation for this seemingly counter-intuitive result is that, below 40–41 °C, DPH is located favorably in the interfacial regions between DPPC bulk domains and small PLFE cluster domains. Laurdan’s GP data as shown in Figure S3 lends support to the formation of DPPC domains in PLFE/DPPC(3:7) archaeosomes. PLFE lipids are negatively charged; thus, due to charge–charge repulsion, small clusters should be energetically more favorable than large PLFE domains. Because the interfacial regions between two distinctly different domains, i.e., monolayer domains of PLFE tetraether lipids and bilayer domains of gel-state DPPC, are likely to possess significant membrane defects, it is conceivable that the rotational hindrance (thus the order parameter) of DPH in the domain interfacial regions is lower than that in DPPC or PLFE liposomal membranes. In the interfacial regions, DPH may undertake a rather slow flip-flop motion [75], in addition to the fast wobbling rotation, thus giving rise to lower R values (Figure 3A). Although the above explanation needs to be tested by more rigorous investigations in the future, it is of note that the rotational rate and the order parameter of DPH in PLFE/DPPC(3:7) archaeosomes do not change with temperature in a continuous and smooth manner, and there is a dip/kink in the curve of R and S versus temperature near 37–41 °C (Figure 3A,B). This also suggests a possible liposomal structural change in PLFE/DPPC(3:7) archaeosomes from 37 to 41 °C, wherein the ZP transition was most prominent (Figure 2).

### 2.4. Effect of Temperature on DXO Release from PLFE/DPPC Hybrid Archaeosomes

**Figure 4:**
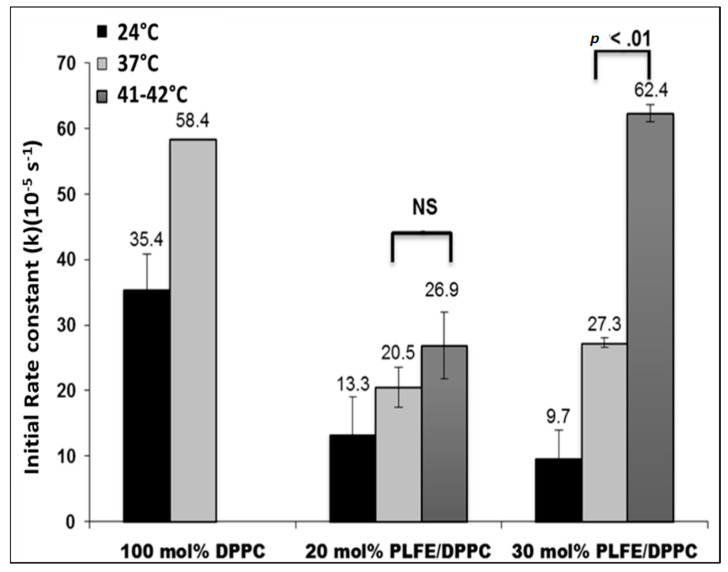
Effects of PLFE content and temperature on doxorubicin (DXO) release from liposomes. DPPC liposomal DXO samples containing 0, 20, and 30 mol% PLFE, with ~200 µM DXO entrapped, were prepared as described in Materials and Methods. The initial rate constant (*k*) was determined by following the enhancement of DXO fluorescence intensity due to spontaneous leakage over time at 24, 37, and 41–42 °C. Liposomes (diameter ~160–200 nm) were dispersed in Tris-HCl buffer (pH 7.2). NS = not significant.

The initial rate constants (*k*) of DXO release from 100 mol% DPPC, 20 mol% PLFE/80 mol% DPPC, and 30 mol% PLFE/70 mol% DPPC was determined at 24 °C, 37 °C, and 41–42 °C. As shown in Figure 4, below the main phase transition temperature (41.5 °C) of DPPC, liposomes with PLFE show a rate constant two times smaller than that in DPPC liposomes without PLFE. The lower rate constant can be attributed to less volume fluctuations due to the membrane ordering effect of PLFE [41,76].

More interestingly, when temperature was increased to 41–42 °C from 37 °C, the rate constant increased dramatically (~2.3 times, a *p* value < 0.01) for PLFE/DPPC(3:7) archaeosomes, but not so dramatically for PLFE/DPPC(2:8) archaeosomes (Figure 4), which correlated well with our ZP data (Figure 2). A sharp ZP transition was observed for PLFE/DPPC(3:7) archaeosomes, but not for PLFE/DPPC(2:8) archaeosomes (Figure 2). The dramatic increase in *k* from 37 °C to 41–42 °C in PLFE/DPPC(3:7) archaeosomes is consistent with our interpretation of the ZP transition, which has been proposed to involve DPPC domain melting and PLFE lipid flip-flop. If these liposomal structural changes indeed occur in PLFE/DPPC(3:7) archaeosomes when the temperature is raised from 37 °C to 41–42 °C, an accompanying dramatic increase in DXO release is expected [76].

**Figure 5:**
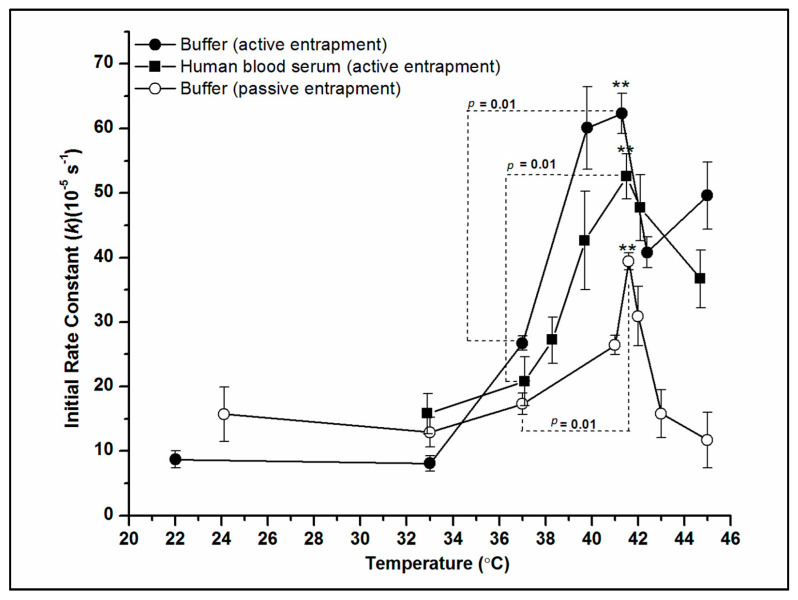
Temperature dependence of the initial rate constant *k* of doxorubicin (DXO) release from PLFE/DPPC(3:7) archaeosomes. Archaeosomes were dispersed either in 50 mM Tris-HCl buffer (pH 7.2) containing 10 mM EDTA and 0.02% of NaN3 or in 50% human blood serum prepared in 10 mM HEPES buffered saline (HBS) containing 145 mM NaCl, 5 mM KCl, 0.1g/L glucose, and 0.1 g/L bovine serum albumin (BSA; pH 7.5). DXO was loaded into liposomes by the active or passive entrapment method as indicated. The *p* values were determined from three samples (*n* = 3).

To further characterize DXO release from PLFE/DPPC(3:7) archaeosomes, we have measured the DXO release rate constant *k* over a wider temperature range and in different media. As shown in Figure 5, *k* is relatively low and insensitive to temperature between 22 and 37 °C. However, there is a ~2.0–2.5-fold sharp increase in *k* from 37 to 41 °C. This abrupt increase in *k* coincides with the ZP transition observed from PLFE/DPPC(3:7) archaeosomes (Figure 2) and is irrespective of the entrapment method (Figure 5). More significantly, this abrupt change in *k* was observed not only in Tris buffer but also in human blood serum (Figure 5), which indicates that the kinetics of DXO release and the physical origin for the abrupt increase in *k* observed from PLFE/DPPC(3:7) archaeosomes by the mild temperature jump from 37 to 42 °C are not affected by serum proteins such as albumin and globulins. These data indicate that PLFE/DPPC(3:7) archaeosomal DXO is potentially useful thermo-sensitive archaeosomal drug and that the mild hyperthermia treatment (i.e., raising the local body temperature to 42–44 °C) commonly used in clinical settings can be employed to trigger controlled drug release from PLFE/DPPC(3:7) archaeosomes.

### 2.5. Interactions of PLFE/DPPC(3:7) Archaeosomal DXO with Live Cells

**Figure 6:**
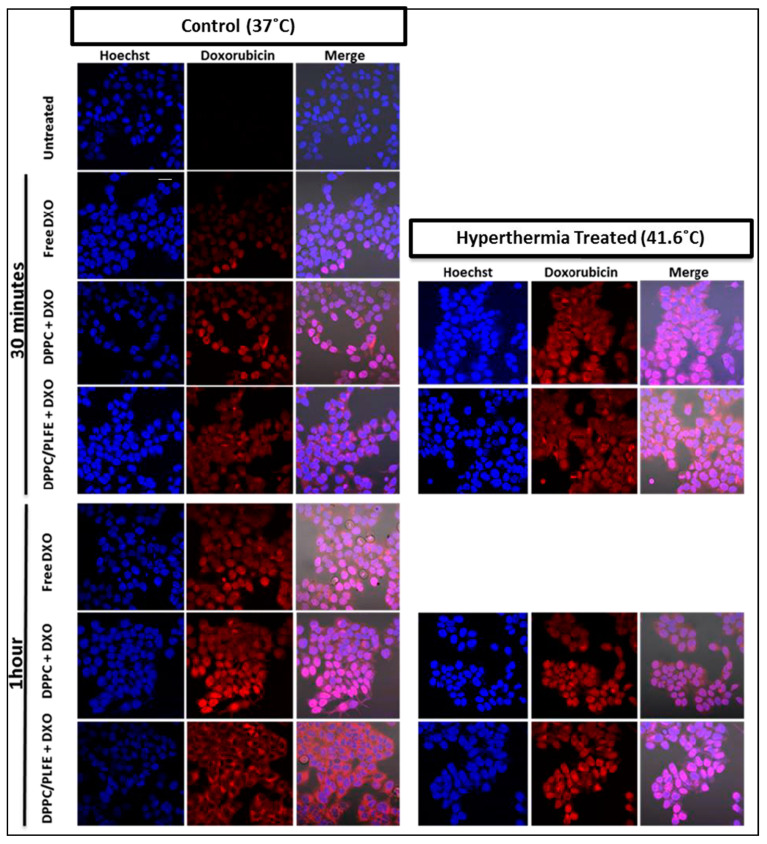
Confocal images of MCF-7 cells treated with free DXO and liposomal DXO with and without hyperthermia treatment. MCF-7 cells treated with 30 nmol of free DXO, DPPC liposomal DXO, or hybrid archaeosomal DXO containing 30 mol% PLFE/70 mol% DPPC for 30 min or 1 h at 37 °C, after a 15 min pre-incubation at 37 °C (control) or 41.6 °C (hyperthermia treatment) (see Figure S4 for the experimental setup). Scale bar = 20 μm.

**Figure 7:**
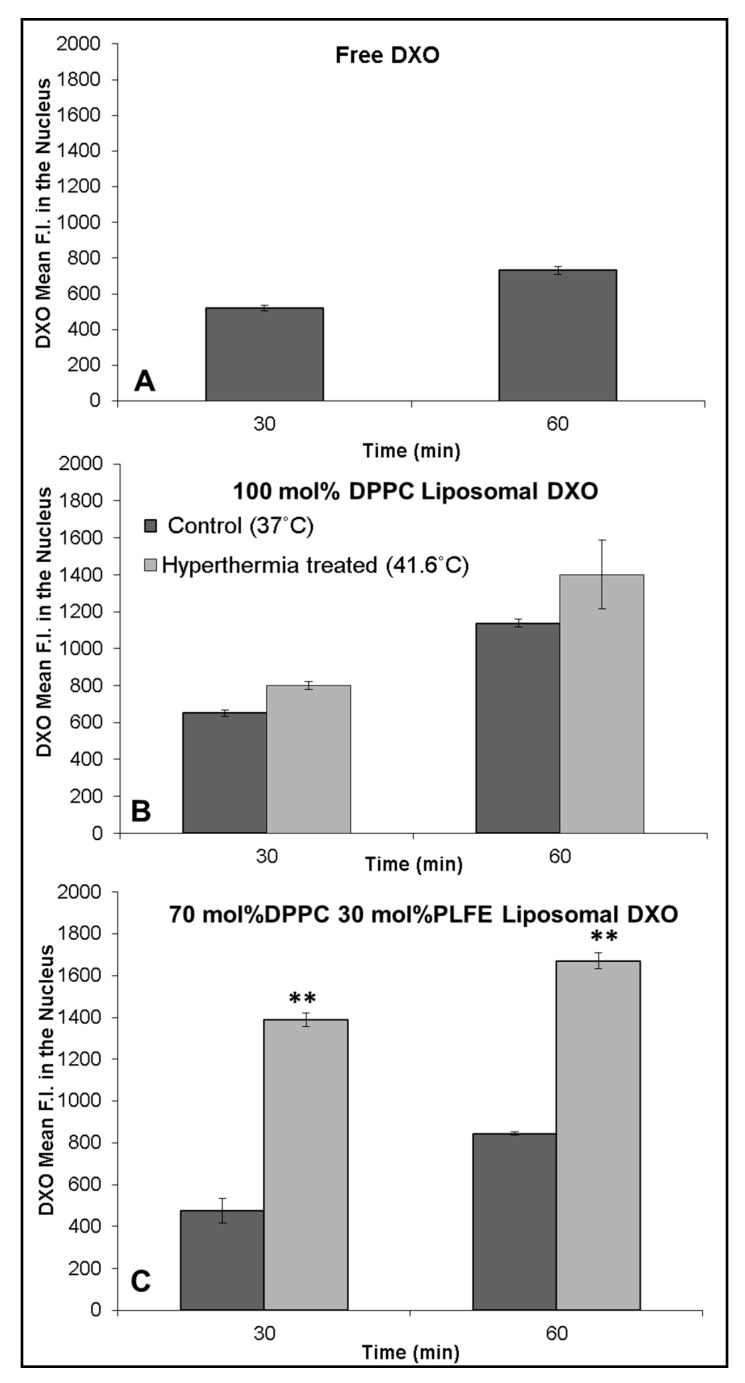
Effect of hyperthermia treatment on DXO’s entering into the nucleus of MCF-7 cells. Fluorescence intensity (F.I.) of DXO in the nucleus of MCF-7 cells was measured after incubation with free DXO (**A**), 100 mol % DPPC liposomal DXO (**B**) and PLFE/DPPC(3:7) archaeosomal DXO (**C**) for 30 and 60 min at 37 °C, with and without the initial 15-min hyperthermia treatment at ~41.6 °C (see Figure S4). F.I. is compared between control (37 °C) and hyperthermia-treated (~41.6 °C) cells. ** *p* < 0.05. Light black and dark black bars in (**C**) bear the same meaning as those in (**B**).

Using fluorescence confocal microscopy, we have evaluated how mild hyperthermia might affect the interactions of PLFE/DPPC(3:7) archaeosomal DXO with live cells. We chose to study the model breast cancer cell line MCF-7 because DXO is widely used to treat breast cancers. Confocal images in Figure 6 show MCF-7 cells treated with free DXO, DPPC liposomal DXO, and the hybrid archaeosomal DXO with 30 mol% PLFE/70 mol% DPPC for 30 min or 1 h at 37 °C after a 15-min pre-incubation at 37 °C (control) or 41.6 °C (mild hyperthermia treatment). The procedures for this experiment are described in detail in Figure S4. Cells were stained with the nuclear dye Hoechst (blue, Figure 6). The fluorescence intensity of DXO (red, Figure 6) in the nucleus of different cells was quantified from confocal images using Zen software, as shown in Figure 7.

The anticancer activity of DXO lies in its intercalation into DNA bases in the nucleus. Thus, it is of interest to compare DXO concentrations in the nuclei when cells are treated with different liposomal DXO under different conditions. Here, we use DXO fluorescence intensity per cell to reflect the relative DXO concentration. At 37 °C, MCF-7 cells treated with free DXO (Figure 7A), DPPC liposomal DXO (Figure 7B), and hybrid archaeosomal DXO with 30 mol% PLFE/70 mol% DPPC (Figure 7C) all showed DXO fluorescence in the nucleus after 30 min and accumulated more after 1 h. This result indicates that, under our experimental conditions, DXO continued to enter the nuclei over the one-hour time course, not yet reaching saturation or degradation stage.

More interestingly, a 15-min hyperthermia treatment (from 37 °C to 41.6 °C) to the mixture of PLFE/DPPC(3:7) archaeosomal DXO and MCF-7 cells increased DXO fluorescence in the cell’s nucleus by ~200% (Figure 7C), but much less (only 14%) in the mixture of DPPC liposomal DXO with MCF-7 cells (Figure 7B). This difference can be explained as follows. First, at 37 °C, DXO release from PLFE/DPPC(3:7) archaeosomes is two times slower than that from DPPC liposomes (Figure 4) due to higher drug retention in the tightly and rigidly packed archaeosomes. Thus, applying hyperthermia treatment can release more DXO from our hybrid archaeosomes than DPPC liposomes. Second, hyperthermia treatment to PLFE/DPPC(3:7) archaeosomes can cause more structural perturbations (e.g., DPPC domain melting and PLFE lipid flip-flop) than hyperthermia treatment to DPPC liposomes (only DPPC melting). An increase in DXO content in the nucleus is highly significant in terms of DXO’s drug efficacy. Hence, the use of PLFE/DPPC(3:7) archaeosomes in conjunction with local hyperthermia treatment (Figure 6 and Figure 7C) could be an effective approach to deliver DXO to targeted tissues.

### 2.6. Cytotoxicity of PLFE/DPPC(3:7) Archaeosomes

**Figure 8:**
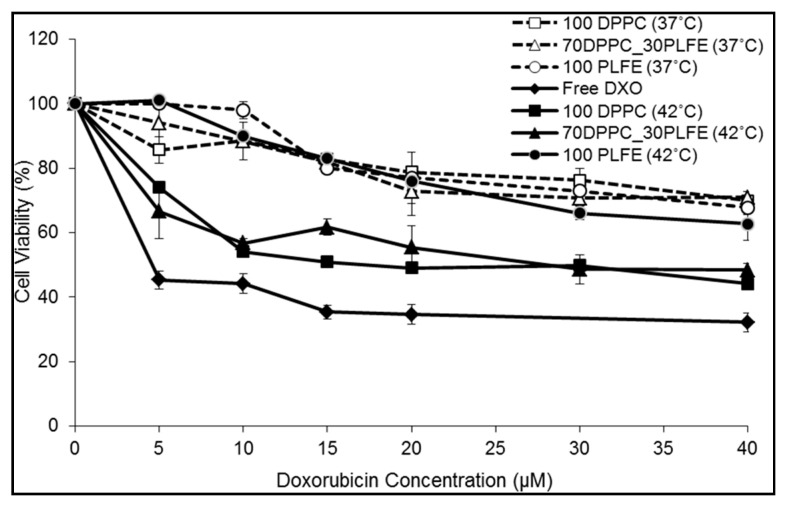
DXO dose dependent cell viability test. MCF-7 cells were treated with various concentrations of liposomal DXO or free DXO. Immediately after treatment, cells were either incubated at 37 °C (control) or 42 °C for 15 min, followed by 24 h incubation at 37 °C. Error bars indicate the standard deviations of three samples.

The CyQuant assay kit was used to monitor the effects of free DXO and various liposomal DXO, with and without mild hyperthermia treatment, on the viability of MCF-7 cells. For MCF-7 cells treated with PLFE liposomal DXO for 24h at 37 °C, there was a moderate and steady decrease in cell viability with increasing DXO dose, dropping to ~70–75% at 40 µM DXO (Figure 8, open and closed circles). An initial 15-min pre-incubation at 42 °C versus 37 °C made no difference to the cytotoxicity of PLFE liposomal DXO. This result is expected because PLFE liposomes do not show a major structural change (e.g., a phase transition or ZP transition) from 37 to 42 °C [41,45,46,47] and exhibit low membrane volume fluctuations between 37 and 42 °C [41].

When treated with PLFE/DPPC(3:7) archaeosomal DXO for 24 h at 37 °C without hyperthermia treatment, cell viability changed only ~25% from 0 to 40 µM DXO (open triangles, Figure 8), similar to the case of PLFE liposomal DXO. In contrast, with hyperthermia treatment (42 °C) for 15 min at the beginning, PLFE/DPPC(3:7) archaeosomal DXO lowered cell viability to ~50% at 40 µM DXO after 24 h incubation at 37 °C (dark triangles, Figure 8). The increased cytotoxicity after pre-incubation at 42 °C can be attributed to hyperthermia-induced drug release resulting from the liposomal structural change involving both DPPC domain melting and PLFE lipid flip-flop, as discussed earlier. The cytotoxicity effect of DPPC liposomal DXO is similar to that of PLFE/DPPC(3:7) archaeosomal DXO. Raising the temperature to 42 °C causes an increase in DXO release from DPPC liposomes due to increased membrane volume fluctuations during the phase transition of DPPC [41], and thus higher cytotoxicity. Figure 8 also shows that DXO entrapped inside liposomes causes less cytotoxicity in comparison to free DXO, which exhibits a 50% inhibition (IC_50_) of cell proliferation at ~4 µM DXO (Figure 8). Untreated cells (as a control) did not show any significant cell death at 42 °C.

In short, the cell proliferation assay demonstrates that PLFE/DPPC(3:7) archaeosomes can serve as a stable thermo-sensitive nano-carrier and that a 15-min incubation at 42 °C (hyperthermia treatment) induces more drug molecules to be released from the liposome, resulting in more cell death.

## 3. Materials and Methods

### 3.1. Archaeal Cells and PLFE Lipids

Cells from the thermoacidophilic archaeon *Sulfolobus acidocaldarious* (American Type Culture Collection (ATCC) #49426, Rockville, MD, USA) were grown aerobically and heterotrophically at 75–80 °C and pH 2.5–3.0, as described [77]. The polar lipid fraction E (PLFE) was isolated from *S. acidocaldarius* dry cells by Soxhlet extraction, thin-layer and column chromatography, and methanol precipitation, as previously described [35,36].

### 3.2. Liposome Preparation

Hybrid liposomes were prepared with PLFE and DPPC (Avanti Polar Lipids, Alabaster, AL, USA). DPPC stock solution was made in chloroform and PLFE stock in chloroform:methanol:water mixture (118:67:15, *v*/*v*/*v*). The concentrations of PLFE and DPPC stock solutions were determined based on the phosphate assay [78]. After pipetting appropriate amounts of DPPC and PLFE into a screw capped Pyrex test tube, the lipids were dried under N_2_ then under vacuum overnight to remove any residual organic solvent. Lipids were hydrated with 50 mM Tris buffer (pH 7.2) containing 10 mM EDTA and 0.02% NaN_3_ (NaN_3_ was omitted for cell studies), followed by vortexing at ~69 °C for ~3 min to make multilamellar vesicles (MLVs). The resulting vesicles were subject to 3 heating (69 °C for 30 min)/cooling (4 °C for 30 min) cycles. Samples were then incubated for 1–2 days at room temperature. Unilamellar vesicles (LUVs, ~200–600 µM) were prepared from MLVs via extrusion (Lipex Biomembranes, Vancouver, BC, Canada) at 69 °C using two stacked polycarbonate membranes (pore size = 200 nm) at ~120 atm N_2_ pressure followed by 1–2 days of incubation at room temperature.

### 3.3. Particle Size and Zeta Potential Measurements

Particle size and size distribution were determined using a Malvern Zetasizer HS spectrometer (Worcestershire, UK). The light source was a 10 mW He-Ne laser (633 nm) and the scattered light was measured at 90° to the incident beam. Zeta potential (ZP) was determined using a Malvern Zetasizer Nano ZS instrument (Worcestershire, UK) based on the electrophoretic mobility measurements through a combination of laser Doppler velocimetry and phase analysis light scattering techniques. The instrument was calibrated using a zeta potential transfer standard (DTS1235, −42 mV, Malvern, Worcestershire, UK). Zeta potential was measured as a function of temperature at a rate of ~0.2 °C/min. Samples were incubated for 10 min at each temperature prior to ZP measurement, and measurements were taken in triplicates at each temperature.

### 3.4. Doxorubicin Entrapment

Doxorubicin (DXO, from Fluka Chemie GmbH, Buchs, Switzerland) was encapsulated into liposomes using either passive or active entrapment methods. For passive drug entrapment, the MLVs were prepared by hydrating dried lipids with 50 mM Tris buffer (pH 7.2) containing 10 mM EDTA and DXO, followed by vortexing at 69 °C and multiple heating/cooling cycles. The MLVs were then extruded as described earlier, followed by three additional heating/cooling cycles.

Liposomal DXO was also prepared using an active entrapment method [79]. In this case, dried lipids were hydrated with 250 mM ammonium sulfate (pH 4). An ammonium sulfate gradient was formed by removing the ammonium sulfate from the external liposome medium by dialysis using a Slide-A-Lyzer dialysis cassette (10,000 MWCO; Pierce, Carlsbad, CA, USA). Specifically, the liposome sample (1 mL containing 500–1000 nmol of lipids) was injected into the dialysis cassette and dialyzed against 300 mL of 50 mM Tris buffer (pH 7.2) containing 140 mM NaCl for 3 h with buffer exchanged every hour. Afterwards, DXO (120–300 µM) was added to the outside buffer at a temperature above the phase transition of the liposomal lipids (e.g., 65 °C for PLFE or PLFE/DPPC mixtures) and incubated for 30 min. An ammonium sulfate gradient was used for loading DXO into liposomes. DXO has a primary amino group with a pK_a_ value of 8.46 [79]. At pH 7.2, ~95% of DXO molecules are in the non-ionized form. The non-ionized form of DXO enters the liposome and is ionized inside the liposome (pH 4) due to the proton-rich environment. The protonated DXO becomes impermeable across the liposomal membrane [80,81]. Un-encapsulated drug was removed using either a Sephadex G-50 column or a Slide-A-Lyzer dialysis cassette against 50 mM Tris buffer (pH 7.2) containing 200 μM NaCl for >5 h.

Drug entrapment was determined after the addition of 0.2% Triton X-100 to lyse the liposome. The absorbance of lysed liposome with DXO was measured at 481 nm using the extinction coefficient of 11,500 M^−1^cm^−1^ (in water) [82]. The percent entrapment was calculated using the following equation: % efficiency = [Liposomal DXO/(Free DXO + Liposomal DXO)] × 100. DXO entrapment efficiency using passive entrapment was <60% whereas the active loading method yielded >90% DXO entrapment in liposomes. The concentration of entrapped DXO in the liposome dispersions was typically ~200 µM for using the active entrapment method and ~100 µM for the passive entrapment method.

### 3.5. Membrane Phase Transition as Assessed by Generalized Polarization of Laurdan Fluorescence

Aliquots of the fluorescent probe 6-lauroyl-1,2-dimethylamino-naphthalene (Laurdan) (Avanti Polar Lipids, Alabaster, AL, USA) in ethanol were added to liposome samples, each of which contained 40 nmol of lipids in a final volume of 1.8 mL (i.e., 22 µM) with a probe-to-lipid ratio of 1/600. The vesicles were incubated with Laurdan at 24 °C for 1 h. The emission spectra of Laurdan fluorescence were measured under gentle stirring at 17–50 °C on an SLM 8000 fluorometer (Urbana, IL, USA). The background readings from vesicles without the probe were subtracted from the sample readings. The generalized polarization (GP = (I_435_ − I_500_)/(I_435_ + I_500_)) was calculated from the spectral readings [83]. Here, I_435_ and I_500_ are the fluorescence intensities measured at 435 and 500 nm, respectively, when the sample is excited at 340 nm. Because Laurdan’s GP values obtained from tetraether lipid membranes cannot be directly compared with those obtained from diester lipid membranes due to the difference in chromophore disposition [47,84] (see Supplementary Materials), in the present study, Laurdan’s GP was not used to monitor membrane packing in PLFE/DPPC mixtures; instead, Laurdan’s GP was used to assess how PLFE content affected the phase behaviors of DPPC liposomes.

### 3.6. Membrane Packing Tightness as Revealed by DPH Anisotropy Decay

To assess liposomal membrane packing tightness, we used differential polarized phase-modulation fluorometry [66,67] to determine the order parameter (S) experienced by the membrane probe 1,6-diphenyl-1,3,5-hexatriene (DPH) (Invitrogen-Molecular Probes, Eugene, OR, USA). DPH stock solution was prepared in methanol and the concentration was determined spectroscopically at 354 nm using the extinction coefficient of 91,000 M^−1^cm^−1^ (in methanol) [69]. DPH in methanol was added into liposome dispersions (0.10–0.15 mM lipids) with the DPH-to-lipid molar ratio of 1/500 and incubated in the fluorometer sample compartment under magnetic stirring at 65 °C for 30 min for PLFE and PLFE/DPPC liposomes or at 50 °C for 1 h for DPPC liposomes, prior to fluorescence measurements.

Samples were illuminated with vertically polarized light and the fluorescence emission was observed through a polarizer and a KV480 cutoff filter. The difference in the phase delay and demodulation ratio between the parallel and perpendicular components of the fluorescence emission was measured on an ISS K2 fluorometer (ISS Inc. Champaign, IL, USA) using a light-emitting diode (LED) at 370 nm as the light source. Differential phase delays and demodulation ratios were measured using 11–15 modulation frequencies ranged from 2 to 100 MHz. The rotational correlation times (θ) and the limiting anisotropy (r_∞_) of DPH in each given membrane system were determined by a multi-exponential anisotropy decay fit using the nonlinear least-squares program provided by ISS (Champaign, IL, USA). The order parameter (S) was calculated by the equation: S^2^ = r_∞_/r_o_ [70], where r_o_, the fundamental fluorescence anisotropy of DPH, was set to 0.39 [71]. The rotational rate R was calculated using the equation R= 1/6θ_1_ [69].

### 3.7. Drug Release

To initiate this experiment, ~50 nmol of liposomal DXO, which was separated from free drug by a Sephadex G-50 column, was rapidly injected into 1.8 mL of the buffer with gentle magnetic stirring in a fluorescence cuvette preincubated at the desired temperature on an SLM 8000 fluorometer (Urbana, IL, USA). Temperature of the sample compartment was controlled by a circulating bath. Slow kinetics of DXO release was monitored in 50 mM Tris buffer (pH 7.2) containing 10 mM EDTA and 200 μM NaCl (equivalent to drug concentration) or in 50% human serum. Blood plasma was obtained from a healthy donor at Temple University Hospital (Philadelphia, PA) dispersed in HEPES buffered saline (HBS, pH 7.5) containing 145 mM NaCl, 5 mM KCl, 0.1 g/L glucose, and 0.1 g/L bovine serum albumin (BSA) (pH 7.5). After removal of coagulating factors, the serum was collected and used for the drug leakage assay. The release of entrapped DXO from liposomes was monitored by measuring the increased DXO fluorescence intensity, due to the relief of self-quenching of DXO fluorescence. Liposomal DXO dispersions were excited at 480 nm, and the fluorescence intensity at 590 nm (F_t_) was recorded as a function of time at a constant temperature on the ISS K2 fluorometer (Champaign, IL, USA). At the end of each experiment, the sample was mixed with 0.2% Triton X-100 to release all the entrapped DXO, providing the maximal fluorescence intensity (F_max_). The equation F = A + B(1 − e^−*kt*^), where *t* is time and A and B are constants, was used to fit the normalized fluorescence intensity F = F_t_/F_max_ [85] to determine the rate constant of leakage *k*. The experiment was repeated at different constant temperatures ranging from 22 to 5 °C.

### 3.8. Mammalian Cell Growth

The MCF-7 (from ATCC) invasive breast ductal carcinoma cell line was grown at 37 °C and 5% CO_2_ with Hyclone Dulbecco’s modified Eagle medium (DMEM) containing high calcium (Fischer, Waltham, MA, USA), supplemented with 10% (*v*/*v*) fetal bovine serum (FBS) (Fischer, Waltham, MA, USA) and 1% (*v*/*v*) penicillin–streptomycin mixture (Cellegro, Manassas, VA, USA). Cells were passaged every 3 days or upon reaching ~80% confluency by trypsinization with 0.25% Trypsin (Fischer, Waltham, MA) and counted using a hemocytometer.

### 3.9. Confocal Microscopy

Approximately 500,000 MCF-7 cells were plated in a Mat-tak glass bottom dish. Then, cells were treated with 30 nmol of free DXO or liposomal DXO and immediately put into incubator sets at either 37°C (control) or 41.6°C (hyperthermia treatment) for 15 min. Afterwards, dishes containing cells with drugs were incubated at 37°C for an additional 30 or 60 min. Nuclear dye, Hoechst 33342, was added to cells 15 min prior to imaging. Treated and stained cells were washed with 1x phosphate-buffered saline (PBS). Images were taken under a Carl Zeiss LSM 510 META confocal microscope (Jena, Germany) with a 40× oil objective and 1.5× digital zoom. DXO was excited with an argon laser at 488 nm and the fluorescence emission was observed through an LP615 filter. Hoechst was excited with a diode laser at 407 nm and the fluorescence centered around 440 nm was observed using an LP420 filter.

### 3.10. Cell Proliferation Assay

The cell proliferation assay was done using a CyQuant kit (Invitrogen, Carlsbad, CA, USA) following the manufacturer’s protocol. Approximately 1000 cells/well in a 96-well plate were plated with 100 µL of cell medium (DMEM with 10% FBS and 1% penicillin–streptavidin) and left to adhere overnight. Cells were then treated with various concentrations of liposomal DXO. After treatment, cells were either incubated at 37 °C (control) or 42 °C for 15 min, followed by 24 h incubation at 37 °C. Treated cells were washed with PBS two times and stored at −70 °C for at least 12 h. Remaining live cells were then quantified using fluorescence intensity measured at 530 nm on a Spectra Max M5 microplate reader (Molecular Devices, Sunnyvale, CA, USA) with excitation at 480 nm. A standard curve was constructed for each sample set using known amounts of MCF-7, and the obtained fluorescence intensities were converted to the number of live cells.

### 3.11. Statistical Analysis

Data were expressed as the mean ± standard deviation. In order to test if there was a statistically significant difference between certain data points, a paired Student’s *t*-test (two tailed) was performed and a *p* value was calculated.

## 4. Conclusions

This study demonstrates that mixing the archaea tetraether lipid PLFE with the diester lipid DPPC at a specific molar ratio (3:7) can generate ‘smart archaeosomes’ with great membrane stability against vesicle coalescence and DXO spontaneous leakage at and below 37 °C (Figure 1, Figure 4 and Figure 5). However, when the temperature is raised to 42–44 °C from 37 °C (mild hyperthermia treatment), this hybrid archaeosome undergoes a drastic change in its surface properties, from highly negatively charged to much less negatively charged, as reflected in the abrupt zeta potential (ZP) transition (Figure 2). We speculate that the sharp ZP transition occurs due to a gross change in the liposomal membrane structure, perhaps involving DPPC domain melting and PLFE lipid flip-flop. Upon this mild temperature perturbation, we detected a significant increase in DXO release from the hybrid archaeosome, an increase in DXO entry into MCF-7 cancer cells’ nuclei, and an increase in cell death. Further *in vitro* investigations may reveal more unique membrane properties of this novel hybrid archaeosome system, elucidate the mechanism underlying the ZP transition, and add a function for targeted delivery to help optimize the formulation for clinical use as an effective delivery system.

Archaea tetraether lipids have been applied to the bloodstreams of animals, which showed no sign of toxicity [65,86,87]. They have high stability in the circulation, thus being promising nano-carriers for therapeutics [52,53,54,65,87,88,89]. In the past, the main obstacle of using archaeosomes for therapeutics delivery has been the lack of an efficient method to trigger the release of entrapped content from the otherwise extremely stable structure. Our current study may provide a solution to this long-standing problem. It is important to note that the archaea tetraether lipids used in the previous animal studies [65,86,87] are not the same as PLFE lipids. For example, Freisleben and his collaborators used the main tetraether phospholipid (TEL) from the archaeon *Thermoplasma acidophilum* to make archaeosomes and demonstrated that TEL archaeosomes were cleared from the circulation in 30 min and accumulated mainly in the liver (80%) and spleen (8%) [86]. Like PLFE, TEL is a group of macrocyclic, asymmetric, bipolar, tetraether lipids. However, the structure of TEL is different from that of PLFE. TEL has phosphoryl glycerol on one of its polar head groups and a gulose sugar moiety on another polar head group. In comparison, PLFE lipids have sugar moieties on both polar head groups and have more sugar moieties per molecule than TEL (Figure S1). Those sugar moieties and the phosphate group can form an extensive hydrogen bonding network on the liposome surface. Since the removal of liposomes by the RES system is decreased with increasing liposome surface hydrophilicity [90], the half-life of PLFE/DPPC hybrid archaeosomes in the circulation is expected to be significantly longer than that of TEL archaeosomes. This idea will be tested by determining the circulation time, biodistribution, pharmacokinetics, and toxicity of PLFE/DPPC(3:7)-DXO in animal models in the future.

## Supplementary Materials

The following are available online at https://www.mdpi.com/1422-0067/21/21/8388/s1 [47,83,84,91].

## Abbreviations

DPH	1,6-diphenyl-1,3,5-hexatriene
DPPC	1,2-dipalmitoyl-*sn*-glycero-3-phosphocholine
DSPE-PEG-2000	1,2-distearoyl-*sn*-glycero-3-phosphoethanolamine-N-[methoxy(poly(ethylene glycol))-2000]
DXO	doxorubicin;
GDNT	glycerol dialkylcalditol tetraether
GDGT	glycerol dialkyglycerol tetraether
Laurdan	6-lauroyl-1,2-dimethylamino-naphthalene
MLV	multilamellar vesicles
MSPC	monostearoylphosphatidylcholine
PLFE	polar lipid fraction E
TSL	thermo-sensitive liposomes
