# Supplementary material for: Polar Lipid Fraction E from Sulfolobus acidocaldarius and Dipalmitoylphosphatidylcholine Can Form Stable yet Thermo-Sensitive Tetraether/Diester Hybrid Archaeosomes with Controlled Release Capability
Source: Int J Mol Sci. 2020 Nov 9;21(21):8388. doi: 10.3390/ijms21218388 (PMC7664881; doi:10.3390/ijms21218388)
Supplement: Supplementary file 1 [file ijms-21-08388-s001.pdf]

Supplementary Materials

Polar Lipid Fraction E from *Sulfolobus acidocaldarius* and Dipalmitoylphosphatidylcholine Can Form Stable yet Thermo-Sensitive Tetraether/Diester Hybrid Archaeosomes with Controlled Release Capability

Umme Ayesa and Parkson Lee-Gau Chong *

Department of Medical Genetics and Molecular Biochemistry, Lewis Katz School of Medicine at Temple University, Philadelphia, PA 19140 USA; tua46021@temple.edu

* Correspondence: pchong02@temple.edu; Tel.: 215-707-4182; Fax: 215-707-7536.

1. Structures of PLFE lipids

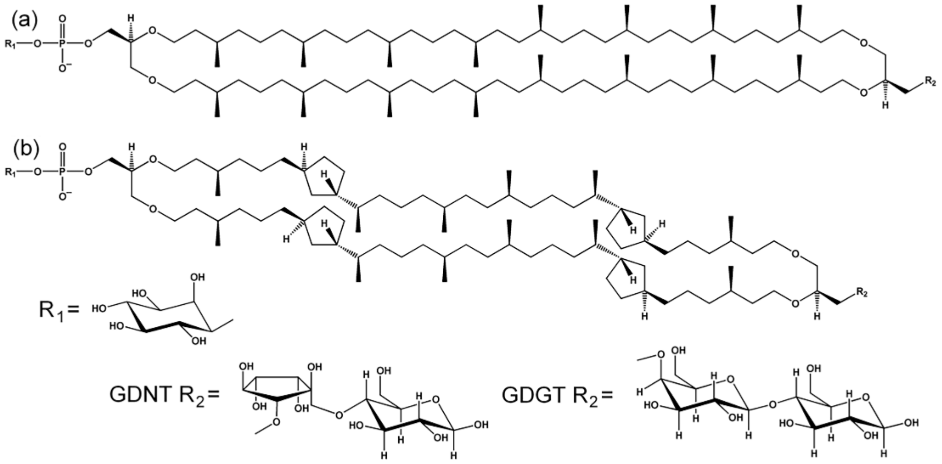

Figure S1. Illustration of structures of lipids in the polar lipid fraction E (PLFE) extracted from the thermoacidophilic archaeon *Sulfolobus acidocaldarius*. There are two components in PLFE: (a) GDGT (glycerol dialky glycerol tetraether) (~10%) and (b) GDNT (glycerol dialky calditol tetraether) (~90%). PLFE lipids are macrocyclic compounds with four tetraether linkages at either end of the structure with two different polar head groups. One of the polar head groups contains phospho-*myo*-inositol (R1), which is negatively charged at neutral pH. The other polar head group is glycerol linked β-D-galactosyl-D-glucose (GDGT R2) or calditol linked β-D-glucose (GDNT R2). Thus, PLFE lipids are asymmetric molecules. In the hydrophobic region, PLFE lipids may contain 0-8 cyclopentene rings in each dibiphytanyl chain. In this illustration, zero and four cyclopentane rings are present in the dibiphytanyl chain for GDGT and GDNT, respectively. Taken from [91] with permission.

__2. Reversibility of the ZP transition__

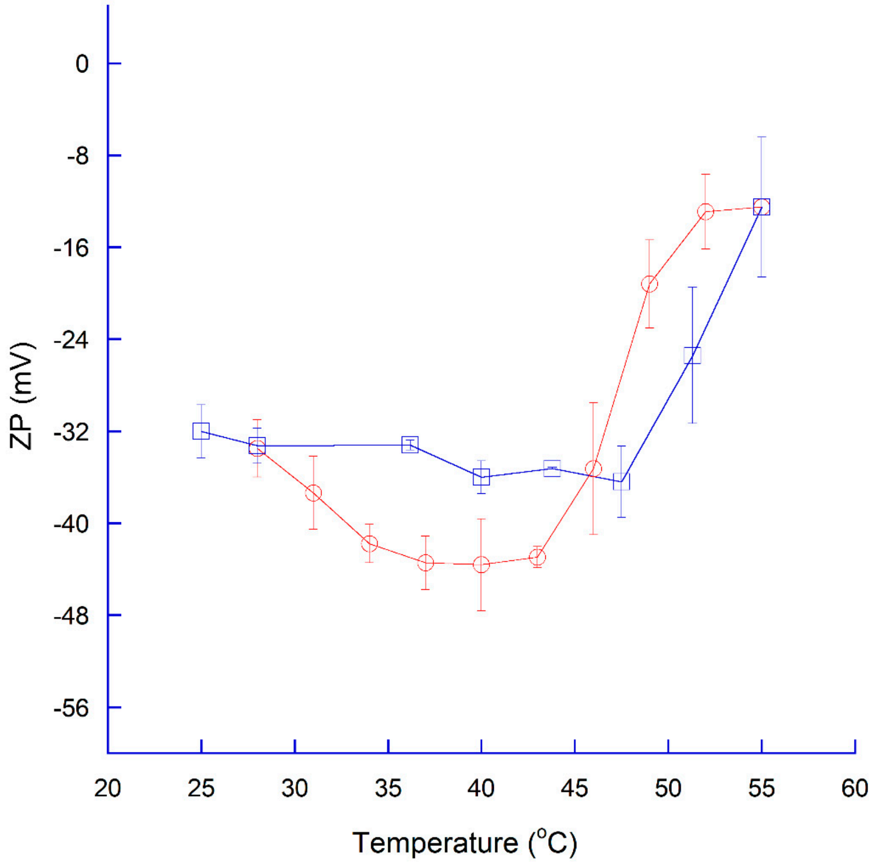

Figure S2. Reversibility of the ZP transition observed from PLFE/DPPC hybrid archaeosomes in 50 mM Tris buffer (pH 7.2) containing 10 mM EDTA and 0.02% NaN_3. A heating scan (red) was followed immediately by a cooling scan (blue). Scan rate ~ 0.2°C/min. The general trend is reversible; however, hysteresis is noticed upon cooling.

3. Generalized polarization (GP) of Laurdan fluorescence

When inserted into diester lipid membranes, the chromophore of Laurdan is located near the lipid polar headgroups and the chromophore's dipole moment is aligned in parallel with the membrane normal [47]. When membrane packing in diester lipid membranes, such as DPPC liposomes, is tight, Laurdan's GP is high, and vice versa [83]. However, the disposition of Laurdan in tetraether lipid membranes, such as PLFE liposomes, is very different from that in diester lipid membranes. In PLFE liposomal membranes, the lauroyl tail of Laurdan inserts into the hydrocarbon core in parallel with the dibiphytanyl chain while the chromophore resides in the lipid polar head group regions with the long molecular axis of the chromophore aligned in parallel with the membrane surface. This unusual L-shape disposition for Laurdan in PLFE liposomes is presumably caused by the rigid and tight packing in PLFE liposomes and by the steric hindrance of the branched methyl group in PLFE lipids. In this disposition, the chromophore of Laurdan in PLFE liposomes is in close proximity to the bound water molecules at the polar head groups and close to the lipid head group polar moieties, and as a result, the degree of "solvent" relaxation is always extensive and the GP value in PLFE liposomes is relatively low at all of the temperatures examined [47]. Because the chromophore disposition is different, Laurdan's GP values obtained from tetraether lipid membranes cannot be directly compared with those obtained from diester lipid membranes [47,84]. For this reason, in this study, GP of Laurdan fluorescence cannot not be used to monitor membrane packing in PLFE/DPPC archaeosomes; instead, it is used to assess how PLFE content affects the phase behavior of DPPC liposomes.

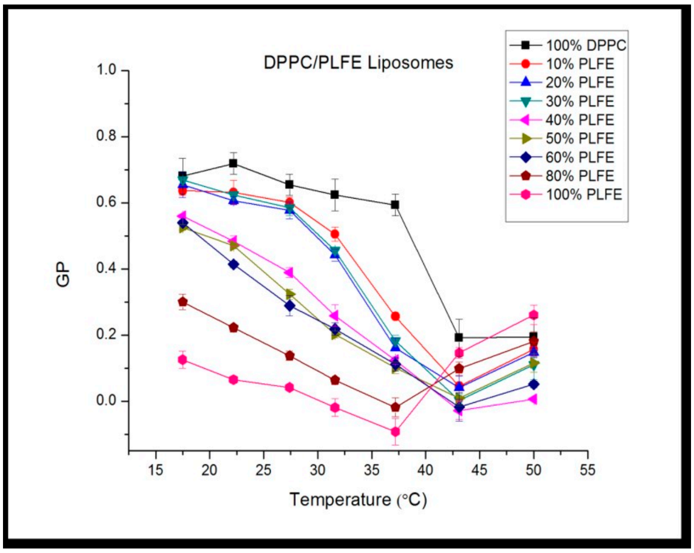

Figure S3. Effect of temperature on the generalized polarization (GP) of Laurdan fluorescence in various PLFE/DPPC archaeosomes with different PLFE mole percents. For liposomes with 100% DPPC, GP drops slightly from 17°C to 37°C, followed by an abrupt decrease from 0.6 at 37.2°C to 0.2 at 43.2°C, with the mid-point

of the abrupt decrease occurred at 40°C, which is close to the known main phase transition temperature 41.5°C of DPPC. For 10-30 mol% PLFE in DPPC, a similar abrupt decrease in GP is seen, although the transition is shifted a little to the lower temperature and not as sharp as that observed in 100% DPPC. This suggests that PLFE acts like cholesterol, attenuating DPPC phase transition. For liposomes containing 40-100 mol% PLFE, there is just a monotonic, not an abrupt, decrease, in GP with increasing temperature, which suggests that above 30 mol% PLFE, there are no sizable DPPC domains in the liposomal membranes.

4. *DPH anisotropy decay parameters analyzed from the differential polarized phase-modulation data*

The measured differential phase delays and demodulation ratios were fitted best with the anisotropy (r) decay equation: $r(t) = r_1 exp(-t/\theta_1) + r_2 exp(-t/\theta_2)$ with θ_2 fixed to a large number such as 10,000 ns. In this case, $r_2 = r_\infty$. The order parameter S was calculated by the equation: $S^2 = r_\infty/r_o$.

Table S1: Analysis of DPH Differential Polarized Phase and Modulation Data

100 mol% DPPC Liposomes

Temperature (°C)	$\theta_{1(ns)}$	R (rad/ns)	r_1	$\theta_2(ns)$	$r_2 = r_\infty$	S	χ^2
25.2	2.26 ± 0.10	0.073 ± 0.003	0.067 ± 0.010	10000 FIXED	0.340 ± 0.001	0.93 ± 0.00	2.89
27.6	2.34 ± 0.30	0.071 ± 0.009	0.063 ± 0.002	10000 FIXED	0.365 ± 0.002	0.96 ± 0.00	2.82
32.3	2.79 ± 0.10	0.069 ± 0.003	0.056 ± 0.000	10000 FIXED	0.344 ± 0.002	0.94 ± 0.00	2.68
35.0	2.17 ± 0.07	0.076 ± 0.002	0.096 ± 0.008	10000 FIXED	0.304 ± 0.002	0.88 ± 0.01	2.24
37.8	1.50 ± 0.10	0.111 ± 0.007	0.136 ± 0.004	10000 FIXED	0.290 ± 0.008	0.86 ± 0.02	1.27
40.5	1.34 ± 0.04	0.124 ± 0.003	0.298 ± 0.005	10000 FIXED	0.120 ± 0.005	0.55 ± 0.01	0.66
42.9	1.28 ± 0.01	0.130 ± 0.001	0.333 ± 0.001	10000 FIXED	0.067 ± 0.001	0.41 ± 0.01	1.74
51.2	1.14 ± 0.04	0.145 ± 0.005	0.400 ± 0.010	10000 FIXED	0.048 ± 0.006	0.32 ± 0.01	2.91

30 mol% PLFE / 70 mol% DPPC Archaeosomes

Temperature (°C)	$\theta_1(ns)$	R (rad/ns)	r_1	$\theta_2(ns)$	$r_2 = r_\infty$	S	χ^2
23.2	4.97 ± 0.40	0.033 ± 0.003	0.090 ± 0.002	10000 FIXED	0.302 ± 0.003	0.88 ± 0.01	2.97
26.7	4.99 ± 0.10	0.033 ± 0.001	0.136 ± 0.001	10000 FIXED	0.240 ± 0.002	0.78 ± 0.00	3.76
30.3	5.05 ± 0.20	0.033 ± 0.001	0.158 ± 0.001	10000 FIXED	0.213 ± 0.003	0.73 ± 0.01	2.38
33.4	4.50 ± 0.10	0.037 ± 0.001	0.197 ± 0.002	10000 FIXED	0.177 ± 0.003	0.67 ± 0.01	2.46
36.3	3.36 ± 0.10	0.050 ± 0.001	0.213 ± 0.001	10000 FIXED	0.169 ± 0.002	0.66 ± 0.00	3.96
39.1	3.65 ± 0.10	0.047 ± 0.001	0.229 ± 0.001	10000 FIXED	0.127 ± 0.002	0.56 ± 0.01	3.76
42.0	3.07 ± 0.09	0.054 ± 0.001	0.247 ± 0.002	10000 FIXED	0.145 ± 0.002	0.61 ± 0.00	2.17
44.9	2.58 ± 0.10	0.064 ± 0.001	0.260 ± 0.003	10000 FIXED	0.130 ± 0.003	0.57 ± 0.01	2.00
48.2	2.15 ± 0.06	0.077 ± 0.001	0.297 ± 0.002	10000 FIXED	0.133 ± 0.002	0.58 ± 0.00	2.41

52.1	2.05 ± 0.09	0.081 ± 0.001	0.312 ± 0.005	10000 FIXED	0.117 ± 0.002	0.55 ± 0.00	1.27

100 mol% PLFE Archaeosomes

Temperature (°C)	$\theta_1(ns)$	R (rad/ns)	r_1	$\theta_2(ns)$	$r_2 = r_\infty$	S	χ^2
24.9	2.73 ± 0.20	0.061 ± 0.002	0.082 ± 0.002	10000 FIXED	0.302 ± 0.002	0.88 ± 0.00	2.66
27.2	3.45 ± 0.30	0.048 ± 0.002	0.088 ± 0.002	10000 FIXED	0.300 ± 0.002	0.88 ± 0.00	1.00
30.0	2.80 ± 0.20	0.059 ± 0.001	0.099 ± 0.002	10000 FIXED	0.290 ± 0.002	0.86 ± 0.00	1.73
32.9	1.92 ± 0.10	0.066 ± 0.001	0.113 ± 0.002	10000 FIXED	0.287 ± 0.002	0.85 ± 0.00	1.92
35.4	2.72 ± 0.10	0.061 ± 0.001	0.121 ± 0.003	10000 FIXED	0.279 ± 0.002	0.84 ± 0.00	1.68
37.8	3.86 ± 0.30	0.043 ± 0.001	0.125 ± 0.002	10000 FIXED	0.262 ± 0.003	0.81 ± 0.01	1.42
41.0	3.11 ± 0.20	0.053 ± 0.002	0.139 ± 0.002	10000 FIXED	0.260 ± 0.002	0.82 ± 0.00	1.91
43.9	3.26 ± 0.20	0.051 ± 0.003	0.156 ± 0.002	10000 FIXED	0.241 ± 0.002	0.79 ± 0.00	1.80
47.2	2.89 ± 0.20	0.057 ± 0.004	0.177 ± 0.002	10000 FIXED	0.229 ± 0.003	0.76 ± 0.01	1.82
50.0	2.80 ± 0.20	0.059 ± 0.004	0.190 ± 0.003	10D00 FIXED	0.217 ± 0.003	0.75 ± 0.01	3.16

5 Experimental design to test how a mild temperature jump from 37°C to 42°C affects the interactions of archaeosomal DXO with MCF-7 breast cancer cells

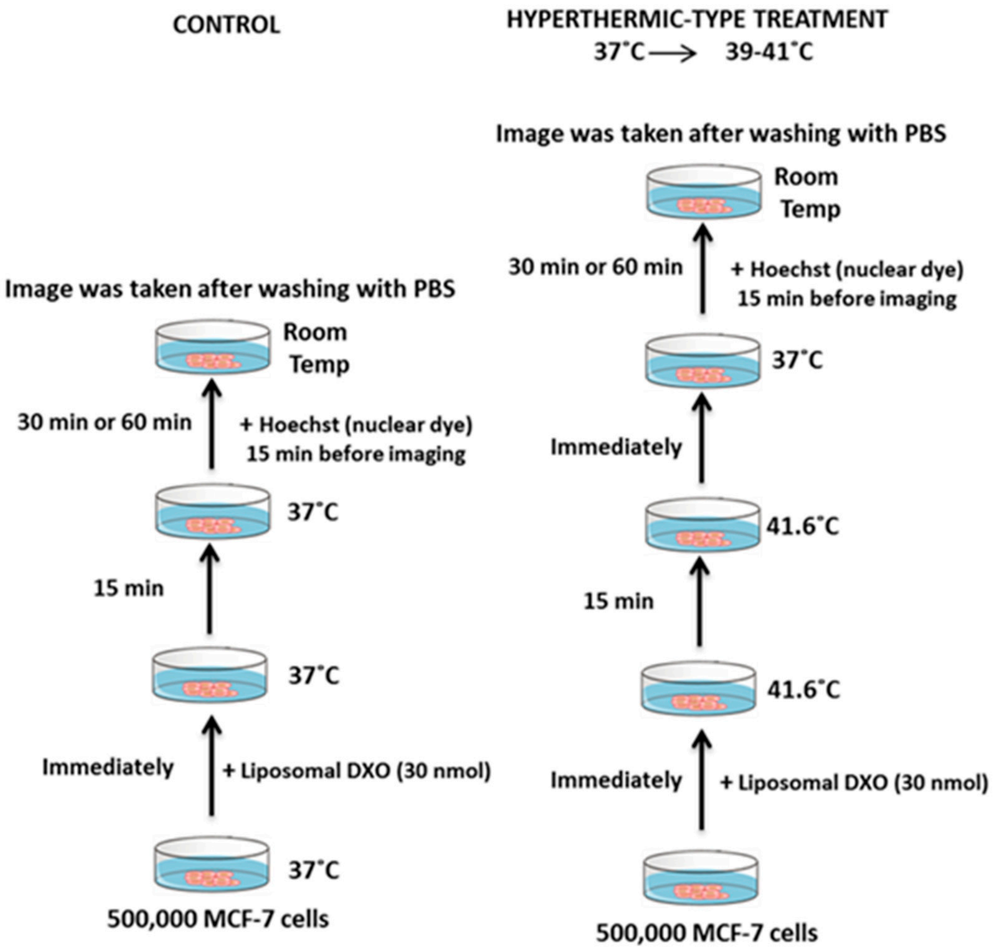

Figure S4. Experimental setup for imaging MCF-7 cells after treatment with free DXO and liposomal DXO for 30 min and 60 min at 37°C, with or without (control) hyperthermic treatment (41.6°C). Approximately 500,000 MCF-7 cells were plated in a Mat-tak glass bottom dish. Then, cells were treated with 30 nmol of free DXO or liposomal DXO and immediately put into an incubator set at either 37°C (control) or 41.6°C (hyperthermia treatment) for 15 min. Afterwards, dishes containing cells with free DXO or liposomal DXO were incubated at 37°C for additional 30 or 60 min. Nuclear dye, Hoechst, was added to cells 15 min prior to imaging. Treated and stained cells were washed with 1x PBS. Images were taken under Zeiss LSM 510 META (Jena, Germany).
